# Cervical, vaginal and vulvar cancer incidence and survival trends in Denmark, Finland, Norway and Sweden with implications to treatment

**DOI:** 10.1186/s12885-022-09582-5

**Published:** 2022-04-26

**Authors:** Kari Hemminki, Anna Kanerva, Asta Försti, Akseli Hemminki

**Affiliations:** 1 Faculty of Medicine and Biomedical Center in Pilsen, Charles University in Prague, 30605 Pilsen, Czech Republic; 2 Division of Cancer Epidemiology, German Cancer Research Center (DKFZ), Im Neuenheimer Feld 580, D-69120 Heidelberg, Germany; 3 Department of Obstetrics and Gynecology, Helsinki University Hospital and University of Helsinki, Helsinki, Finland; 4 Cancer Gene Therapy Group, Translational Immunology Research Program, University of Helsinki, Helsinki, Finland; 5 Hopp Children’s Cancer Center (KiTZ), Heidelberg, Germany; 6 Division of Pediatric Neurooncology, German Cancer Research Center (DKFZ), German Cancer Consortium (DKTK), Heidelberg, Germany; 7 Comprehensive Cancer Center, Helsinki University Hospital, Helsinki, Finland

**Keywords:** Incidence trends, Human papilloma virus, Risk factors, Age-specific incidence, Relative survival

## Abstract

**Background:**

Incidence of cervical cancer has been reduced by organized screening while for vaginal and vulvar cancers no systematic screening has been implemented. All these cancers are associated with human papilloma virus (HPV) infection. We wanted to analyze incidence trends and relative survival in these cancers with specific questions about the possible covariation of incidence, survival changes coinciding with incidence changes and the role of treatment in survival. We used nationwide cancer registry data for Denmark (DK), Finland (FI), Norway (NO) and Sweden (SE) to address these questions.

**Methods:**

We use the NORDCAN database for the analyses: incidence data were available from 1943 in DK, 1953 in FI and NO and 1960 in SE, through 2016. Survival data were available from 1967 through 2016. World standard population was used in age standardization.

**Results:**

In each country the incidence of cervical cancer declined subsequent to rolling out of screening activities. The attained plateau incidence was lowest at 4/100,000 in FI and highest at 10/100,000 in DK and NO. The incidence of vaginal and vulvar cancer remained relatively constant at about 2/100,000. Relative 1-year survival in cervical cancer improved in all countries from low 80%s to high 80%s in the 50-year period, and 5-year survival improved also but at 20% units lower level. Survival gains were found only in patients diagnosed before age 60 years. Survival in vaginal and vulvar cancer followed the same patterns but at a few % units lower level.

**Conclusion:**

Cervical cancer screening appeared to have reached its limits in the Nordic countries by year 2000. Novel treatments, such as immunotherapy, would be needed to improve survival until HPV vaccination will reach population coverage and boost the global fight against these cancers.

## Background

More than half million cervical cancers are annually diagnosed in the world, a large majority of them in developing countries where cervical cancer ranks second after breast cancer [1]. Although human papilloma virus (HPV) is the major risk factor also for vaginal cancer, its global incidence rates differ from that of cervical cancer, and many developed countries have rates at the level of developing countries [1, 2]. Vaginal cancer is rarer than vulvar cancer in most countries and its association with HPV is much higher than that of vulvar cancer [2, 3]. The Nordic countries, Denmark (DK), Finland (FI), Norway (NO) and Sweden (SE), implemented national cervical cancer screening programs relatively early, FI in 1971, SE in 1973, NO 1995 and DK in 1997 and attendance rates have been generally high (details of screening programs are given under Materials and Methods) [4, 5]. Moreover, regional screening was started even before the national one [4, 6]. Incidence trends for cervical cancer markedly decreased subsequent to the implementation of screening activities [7, 8]. The experience from cervical cancer screening has shown that sensitivity of detection is highest among older patients (in their 50s) and tumors of advanced stage [9]. In contrast, no similar decrease has been observed in incidence for vaginal and vulvar cancer consistent with the view that cervical cancer screening is not effective in detecting these rare cancers probably in part because the resources are allocated to cervical screening [1, 7]. Other risk factors for cervical cancer include immune suppression, smoking, family history, germline genetics and reproductive factors [10–15]. Immune suppression and family history are also risk factors for vaginal and vulvar cancer [12, 13, 16].

Therapy for cervical, vaginal and vulvar cancers is multimodal (https://www.esmo.org/guidelines/gynaecological-cancers) [10]. Surgery is the main treatment for local cervical cancer [10, 17]. Concomitant chemoradiotherapy with high dose rate brachytherapy is preferred for large or locally advanced tumors. Chemotherapy is used in advanced tumors either as a neoadjuvant regimen or a palliative treatment. Disease recurrences are often treated with chemotherapy, but pelvic exenteration is a salvage procedure performed for centrally recurrent cervical cancer. SE national treatment patters have been summarized stating that further optimization is needed for stage III-IVA cervical cancers [18]. New treatments with targeted medicines may include bevacizumab and immune checkpoint inhibitors, but as the present follow-up period ended in 2016, targeted treatments had not yet been implemented [10]. For vaginal and vulvar cancer, many similar treatment options are available (https://www.esmo.org/guidelines/gynaecological-cancers) [19, 20]. Early-stage tumors may be surgically treated with possible adjuvant radiation. Many vaginal tumors are unresectable, and are mostly treated with chemoradiation [20]. For vulvar cancer, loco-regional treatment is designed depending on the extent of disease spread into the vulva and in the regional lymph nodes [21]. Minimally invasive tumors may be treated with local excision alone but all other tumors require additionally inguinal lymph node dissection or radiation therapy, and postoperative radiation therapy may be used [21]. Prognosis tends to be better in HPV-related cases [19]. An earlier survival study from the Nordic countries observed improved survival only in the younger age groups [7]. A global survival study reported large regional differences but only slight temporal improvements [22].

In the present analysis we compared incidence and survival trends in cervical, vaginal and vulvar cancer in the Nordic countries which are unique in starting nation-wide cancer registration before other countries, Denmark (DK) in 1943, Finland (FI) and Norway (NO) in 1953 and Sweden (SE) in 1958 [23]. Other features from these countries include high-level medical care and essentially free-of-charge population access, which should ensure ‘real world’ outcome data for these cancers. Health care system in these countries have been quite similar, including also the relative funding; health care expenditure of the gross national income has been over 7% in FI and SE and over 8% in DK and NO (year 2000 from https://www.macrotrends.net/countries/). However, there is a difference between rich NO, spending $2949/capita, and the poorer FI, affording $1723/capita. The specific questions are how the uptake of screening influenced the national trends in theses cancer, how it affected survival and to what extent treatment might have influenced survival. While the literature on cervical cancer screening has focused on cause-specific mortality, survival trends have attracted less attention.

## Materials and methods

We used the NORDCAN database, which is a compilation of data from the high-level Nordic cancer registries as described [24] (https://NORDCAN.iarc.fr/en/database#bloc2). In the database, cervical cancer is covered by ICD10 code C53 and combined vaginal and vulvar cancers by codes C51 (vulva), 52 (vagina) and C57.7–9 (other, multiple or unspecified localizations); all histologies are included without histological specifications. For cervical cancer squamous cell histology has been the main type but adenocarcinoma has been increasing to about 20% of all [25]. In SE adenocarcinoma accounted for 23% of vaginal and 9% of vulval cancers during 1958 to 2004 [26]. Follow-up was to the end of year 2016.

For incidence analysis, the world standard population was used in age adjustment. In incidence diagrams, 5-year smoothing was used because of small case numbers. As a consequence, in the figures showing incidence trends, the first and the last data point is in the middle of the first and last 5-year period. For cervical cancer, age-specific incidence data were presented.

Survival data for relative survival were available from 1967 onwards and the analysis was based on the cohort survival method for the first nine 5-year periods from 1964 to 2011, and a hybrid analysis combining period and cohort survival in the last period 2012–2016, as detailed [27, 28]. The hybrid method includes cases from the penultimate 5-year period to allow for a 5-year survival [29]. Age groups 0 to 89 were considered, and for age-standardization the International Cancer Survival Standard was used. The country-specific life tables were used to calculate the expected survival. Age-specific survival figures are also shown.

In addition to the survival figures we calculated period-specific differences between 1- and 5-year survival as an indicator of how survival changed between years 1 and 5 after diagnosis. If the difference remains constant over time, no improvement took place in this interval; if it decreased survival improved in this interval.

Aggregated data from a publicly accessible database were used posing no ethical issues. Hence no ethical review application was submitted. We confirm that all methods were carried out in accordance with relevant guidelines and regulations.

### Cervical cancer screening programs

National cervical cancer screening programs were implemented in FI in 1971, SE in 1973 (Gothenburg in 1977), NO 1995 and DK in 1997 but regional screening started earlier [5]. Screening activities started in DK in in 1962 (some 40% of the population was covered in 1967) and in FI in 1963, and these were stepwise rolled out to a national coverage [4]. Similarly, the start in NO was 1959 and in SE in 1967 [25]. In addition to the organized screening, opportunistic screening took place, particularly in NO. [4] The participation rates have range from low 70% in NO, to intermediate 75% in DK and 83% in SE, and to high 93% in FI [5]. The participation figures are for year 1997 (or NO 2000) but the attendance rates have fluctuated and for example in FI these have decreased in favor of opportunistic screening [30]. The starting age has been 25 years in FI with 5 year screening frequency; in the other countries the starting age has been 23/25 years and screening frequency 3 years. NO has recommended screening up to 69 years, 10 years longer than the other countries [5]. Screening has been free of charge in countries other than SE where charges have depended on the county of residence.

## Results

**Fig. 1:**
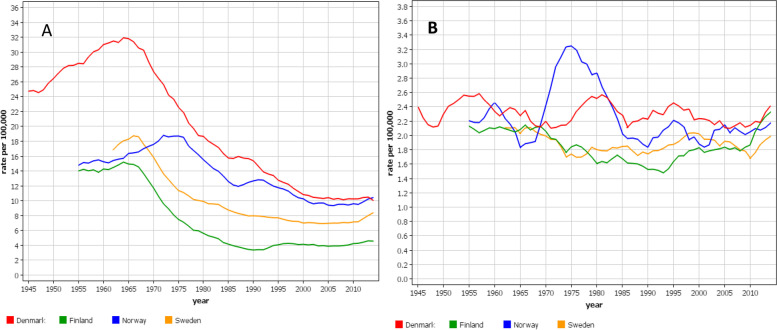
Age-standardized incidence trends for cervical (**A**) and vaginal and vulvar (**B**) cancers from Denmark, Finland, Norway and Sweden (5-year smoothing)

Cervical cancer patient numbers ranged from 12,609 (FI) to 33,457 (SE) between years 1960 and 2016. For vaginal and vulvar cancer the range was from 5384 (FI) to 10,252 (SE); the median diagnostic ages were 72 years for DK and NO, and 73 years for FI and SE. Age-standardized incidence rates for cervical cancer are shown in Fig. 1A. There was an initial maximum for each country, the level of which was highest in DK. The maximal incidence was reached in DK and FI in 1964, in SE 1967 and in NO in 1973. The maximum was followed by a steep decline in incidence which in DK and FI was stabilized at 25–30% of the initial level. The time to reach the plateau took 25 years in FI, 30 years in NO, 35 year in SE and 45 years in DK. The incidence at plateau was highest in DK and NO (10/100,000) and lowest in FI (4/100,000). After 2010 a modest increase in incidence was observed in countries other than DK. The rates for vaginal and vulvar cancer are shown in Fig. 1B. They showed modest U-shaped trends for FI and SE, and even rate for DK. No covariation with cervical cancer rates was apparent. In the final period the incidences were approximately equal in each country (2/100,000). The solitary peak in NO at around 1975 may be related to a temporary change in recording practice; this peak was not present in Norwegian data on gynecological cancers with morphological confirmation suggesting undefined misclassification in our data from that period [31].

**Fig. 2:**
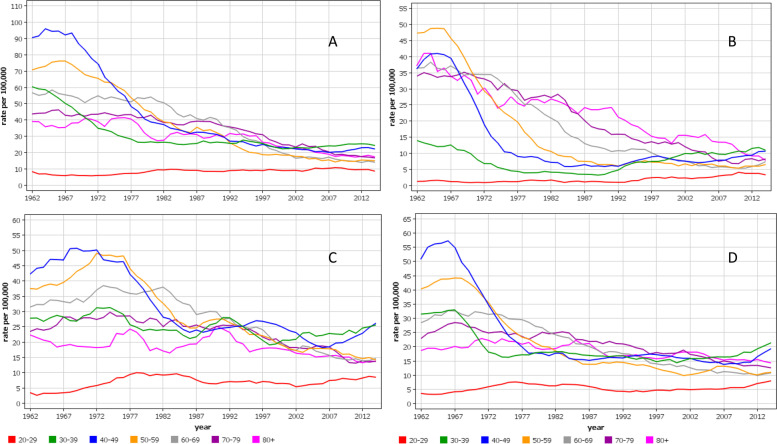
Age-specific incidence trends for cervical cancer from Denmark (**A**), Finland (**B**), Norway (**C**) and Sweden (**D**) (5-year smoothing). Note the differences in y-axes

Age-group specific analysis for cervical cancer showed that the early peak incidence was highest among 40–49 year old women with the exception of FI with the highest incidence among 50–59 year old women (Fig. 2). The peaks at around 1965 were narrow for FI and SE 40–59 year old women while they were wider for DK and NO women, and for NO they occurred before and after 1970 depending on the age group. Another notable point is that the increasing incidence towards the end of the follow-up was limited to 30–49 year old women, which particularly in NO deviated from the declining rates of the old women.

**Fig. 3:**
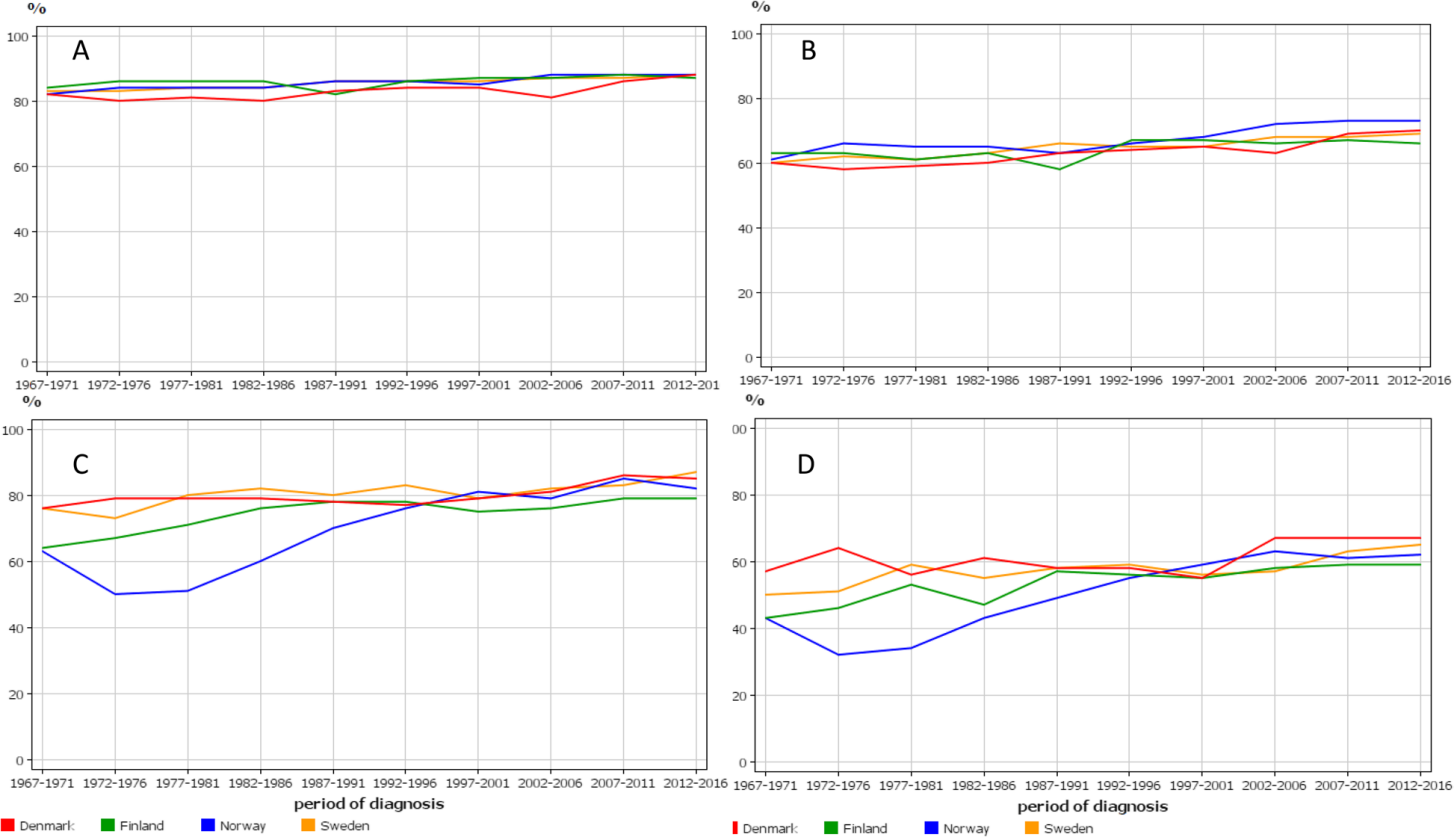
Relative 1-year (**A, C**) and 5-year (**B, D**) survival for cervical cancer (**A, B**) and vaginal and vulvar cancer (**C, D**)

Relative 1-and 5- year survival is shown in Fig. 3 for cervical (A and B) and vaginal and vulvar (C and D) cancers. For cervical cancer, the national rates were practically superimposable, 1-year survival improving from low 80%s to high 80%s in the 50-year period; for 5-year survival the increase was from low 60%s to about 70%. While 1-year survival appeared to increase linearly, 5-year remained flat to about 1990 and increased thereafter. For vaginal and vulvar cancer, the early rates for NO were poor but by year 2000 these caught up with the others. FI rates were also low and after year 2000 they were the lowest, 1-year survival reaching 79% in the final period, compared to 82 to 87% for the others.

Detailed survival figures with 95% confidence intervals are found in Tables 1 and 2. Additionally we show the differences (shown as Diff in the bottom parts of the tables) between 1-and 5-years survival percentages as an indication if any improvement had taken place between years 1 and 5. For NO the improvement was largest, from 21 to 15% (6% units); for FI there was no clear improvement (Table 1). For countries other than FI, most of the positive development was observed after 1997. For the rare vaginal and vulvar cancers the periodic variation was large but the conclusion was that, with the small exception of SE, survival between years 1 and 5 had not improved (Table 2).

**Table 1:** Relative 1- and 5-year survival % with 95% confidence intervals and their difference (Diff) in cervical cancer in the Nordic countries

**1-y survival**	**Denmark**	**Finland**	**Norway**	**Sweden**
1967–1971	82 [80;83]	84 [82;86]	82 [80;84]	83 [81;84]
1972–1976	80 [79;82]	86 [84;88]	84 [82;86]	83 [82;85]
1977–1981	81 [80;83]	86 [84;88]	84 [82;86]	84 [82;85]
1982–1986	80 [79;82]	86 [84;88]	84 [82;86]	84 [83;86]
1987–1991	83 [82;85]	82 [79;85]	86 [84;88]	86 [84;87]
1992–1996	84 [83;86]	86 [83;88]	86 [84;88]	86 [84;87]
1997–2001	84 [82;86]	87 [84;89]	85 [83;87]	86 [84;87]
2002–2006	81 [79;83]	87 [84;90]	88 [87;90]	87 [85;88]
2007–2011	86 [84;88]	88 [85;91]	88 [86;90]	87 [86;89]
2012–2016	88 [87;90]	87 [85;89]	88 [86;90]	88 [86;89]
**5-y survival**				
1967–1971	60 [58;62] 22	63 [60;66] 21	61 [59;64] 21	60 [58;61] 23
1972–1976	58 [56;60] 22	63 [60;66] 23	66 [63;68] 22	62 [60;63] 21
1977–1981	59 [57;61] 22	61 [58;65] 25	65 [62;67] 21	61 [59;63] 23
1982–1986	60 [58;62] 20	63 [59;66] 23	65 [62;67] 19	63 [61;65] 21
1987–1991	63 [61;65] 20	58 [54;62] 24	63 [61;66] 23	66 [64;68] 20
1992–1996	64 [62;66] 20	67 [63;70] 19	66 [64;69] 20	65 [63;67] 21
1997–2001	65 [62;67] 19	67 [64;71] 20	68 [66;71] 21	65 [63;67] 21
2002–2006	63 [61;65] 18	66 [63;70] 21	72 [69;75] 16	68 [66;70] 19
2007–2011	69 [66;71] 17	67 [63;71] 21	73 [70;75] 15	68 [66;70] 19
2012–2016	70 [68;72] 18	66 [63;70] 21	73 [70;75] 15	69 [67;71] 19

**Table 2:** Relative 1- and 5-year survival % with 95% confidence intervals and their difference (Diff) in vaginal and vulvar cancer in the Nordic countries

**1-y survival**	**Denmark**	**Finland**	**Norway**	**Sweden**
1967–1971	76 [72;81]	64 [59;70]	63 [58;68]	76 [73;79]
1972–1976	79 [76;83]	67 [62;72]	59 [46;55]	73 [69;76]
1977–1981	79 [75;82]	71 [67;77]	51 [47;56]	80 [77;83]
1982–1986	79 [75;82]	76 [84;88]	60 [55;65]	82 [79;84]
1987–1991	78 [75;82]	78 [74;82]	70 [66;78]	80 [77;83]
1992–1996	77 [74;81]	78 [74;83]	76 [72;80]	83 [80;86]
1997–2001	70 [75;82]	75 [71;79]	81 [77;85]	79 [76;81]
2002–2006	81 [78;84]	76 [72;80]	79[75;83]	82 [79;84]
2007–2011	86 [83;89]	79 [76;83]	85 [81;88]	83 [81;86]
2012–2016	85 [83;88]	79 [76;81]	82 [78;85]	87 [85;89]
**5-y survival**				
1967–1971	57 [51;63] 21	43 [37;49] 21	43 [37;49] 20	50 [46;54] 26
1972–1976	64 [59;69] 15	46 [40;53] 21	32 [28;37] 18	51 [47;56] 22
1977–1981	56 [52;61] 23	53 [47;60] 18	34 [30;39] 17	59 [55;63] 21
1982–1986	61 [56;66] 18	47 [42;53] 29	43 [38;48] 17	55 [51;59] 27
1987–1991	58 [53;63] 20	57 [52;63] 21	49 [43;55] 21	58 [54;62] 22
1992–1996	58 [54;63] 19	56 [51;62] 18	55 [49;60] 21	59 [55;63] 24
1997–2001	55 [50;60] 24	55 [50;61] 20	59 [54;65] 22	56 [53;60] 23
2002–2006	67 [63;72] 14	68 [53;63] 18	63 [58;67] 16	57 [54;61] 25
2007–2011	67 [63;72] 19	59 [55;64] 20	61 [56;66] 24	63 [59;66] 20
2012–2016	67 [63;70] 19	59 [55;62] 20	62 [58;66] 20	65 [62;68] 22

**Fig. 4:**
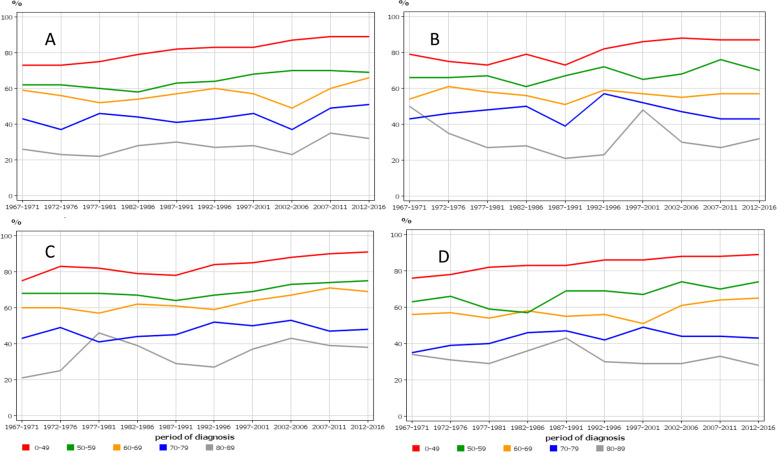
Age-specific 5-year relative survival trends for cervical cancer from Denmark (**A**), Finland (**B**), Norway (**C**) and Sweden (**D**)

Age-specific 5-year survival trends in cervical cancer were similar between the Nordic countries, showing improvements only in those diagnosed before age 60 years (Fig. 4). The consequence of this development was that the survival gap between the young and old patients increased over time.

**Fig. 5:**
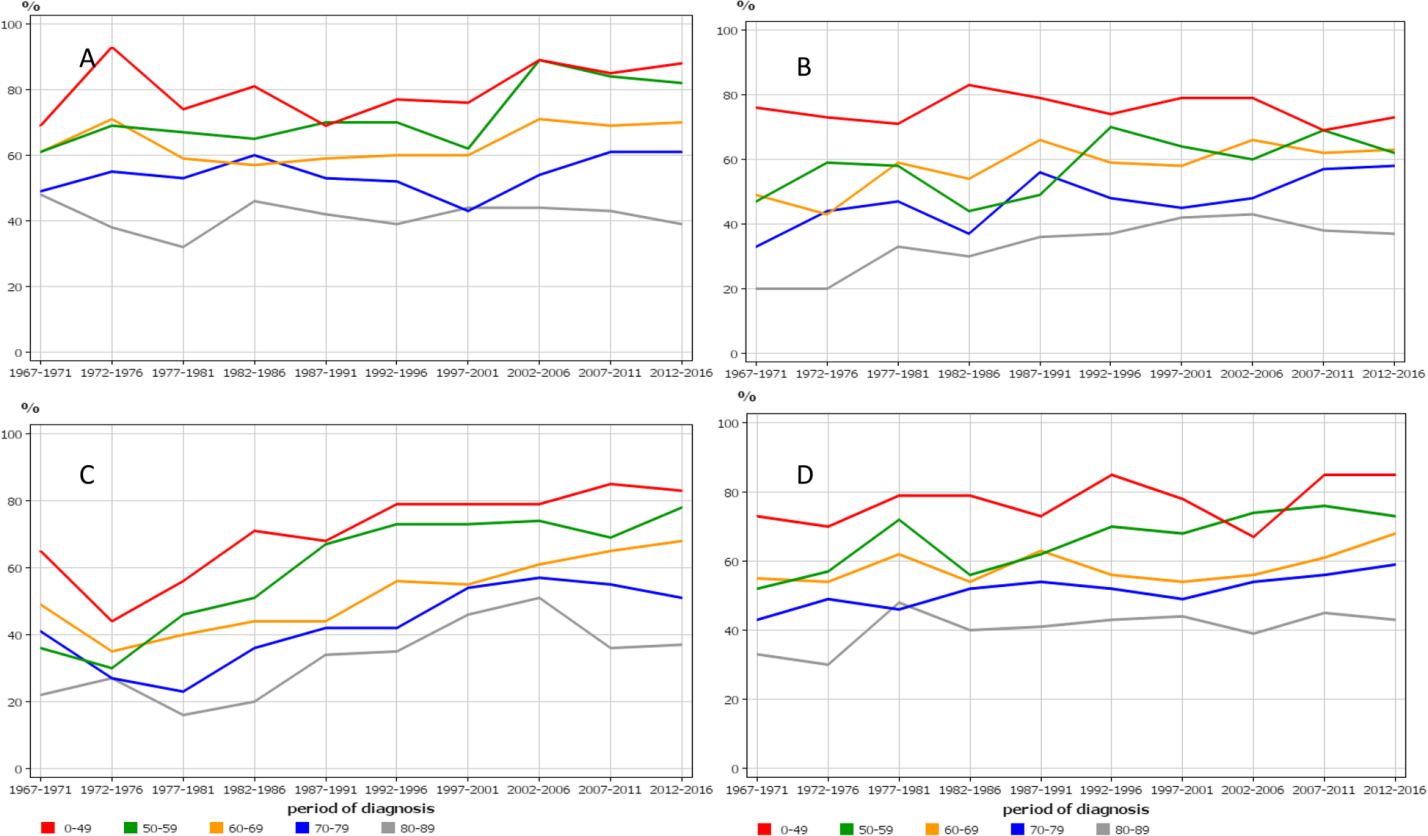
Age-specific 5-year relative survival trends for vaginal and vulvar cancer from Denmark (**A**), Finland (**B**), Norway (**C**) and Sweden (**D**)

Age-specific 5-year survival trends in vaginal and vulvar cancer followed a similar pattern (Fig. 5). Only survival in NO showed improvements in all age groups but the NO starting level was below that of other countries.

As reference to the above survival rates we analyzed survival in all female cancers in NORDCAN (excluding non-melanoma skin cancer) between the first (1967–71) and last (2012–16) 5-year periods. In DK the increase in 1-year survival was from 56 to 81%, FI 55 to 81%, NO 58 to 82% and SE 60 to 83%. In 5-year survival the rates were DK 37 to 64%, FI 34 to 67%, NO 38 to 67% and SE 41 to 67%. From these data covering a 50-year period one can calculate that survival difference between years 1 and 5 improved by 2% units in DK, 7% units in FI, 5% units in NO and 3% units in SE.

## Discussion

Cervical cancer is considered a prime example of successful prevention through screening [5, 32–35]. In our Fig. 1 the large decrease in incidence coincided with the implementation of regional screening programs and was completed when national screening programs were established in the Nordic countries, attesting to the preventive potential of cervical cancer screening. The time difference between the peak and subsequent incidence plateau was shortest in FI (25 years) and longest in DK (45 years), probably indicating the time to reach maximal country-specific coverage of screening. Another influence of screening has been the lowering of diagnostic age of cervical cancers and the incidence was highest among 30–39 year old women towards the end of the follow-up. In some countries, particularly in NO, the incidence showed an increase in the young age groups [25]; in NO increase in cervical cancer incidence has been reported even at age group below 30 years [36]. The reasons for the increase is not known but HPV prevalence is highest in age groups 14 to 24 years; whether HPV-testing may lead to a temporary increase in detected cancers could be an explanation [25]. The results additionally show that the current screening of cervical cancer does not eliminate this cancer for various reasons, such as selective attendance [37, 38]. The participation rates in screening correlate with the most recent incidence rates in cervical cancer, FI has the lowest incidence with 93% participation and DK and NO have the highest incidence with 75 and 70% participation [5]. HPV vaccination is becoming another arm in fighting cervical and other HPV-related cancers but its influence will take some time [39–41]. Figure 1 shows also that screening did not influence risk of vaginal and vulvar cancer, most of which are diagnosed past the cervical cancer screening age (median diagnostic age in Sweden 73 years) [16]. While cervical cancer incidence was declining compared to the relatively constant incidence for vaginal and vulvar cancer, the incidence ratio between these cancers narrowed to 2 [cervix/(vagina+vulva)] in FI and 5 in DK; at the cervical cancer peak year of 1965, the ratios were 7 for FI and 13 for DK. Italian data on vulvar cancer has shown a declining trend in old but an increasing trend in young women [42].

The present results showed modest improvements in survival of cervical, vaginal and vulvar cancers. Admittedly, a 5% unit survival improvement in 1-year survival is a step forward when survival rate is about 80% at the baseline, as for cervical cancer. However, the drop to 60% in the following 4 years appears to indicate lasting difficulties in curing non-localized tumors. The survival age gaps were large; in cervical cancer diagnosed at age over 70 years, 5-year survival did not improve over the study period and thus the age gap increased with time. At the final period the survival gap between the oldest and youngest age groups was close to 3-fold. In vaginal and vulvar cancer, 5-year survival even among the youngest patients did not appear to have make consistent improvements.

Large incidence changes generally pose a warning signal to the interpretation of survival data, particularly, if the causes for the change are not known [43]. In case of cervical cancer, the large incidence changes were attributed to the uptake of screening in the population. Screening-detected cancers are found earlier than symptomatic cancers which as such may lead to improvement in survival. However, in cervical cancer the situation is more complex in the implementation phase because screening is particularly effective in detecting advanced stage cancers [9, 44, 45]. Thus we have to be careful in judging the possible role of treatment in influencing survival in cervical cancer. However, considering that screening was extended only to ages 59 to 69 years, depending on the country, and that no essential improvement in survival was seen in women diagnosed past age 60 years, treatment may have contributed to the observed survival gains only in younger age groups.

To put the survival data in context with all female cancers (minus non-melanoma skin cancer), cervical cancer 1-year survival was far better than that for all cancer in 1967–71, about 82% compared to 55–60% (depending on the country) for all cancer. This was also true of 5-year survival, 61% compared to 34–41% for all cancer. However, in 2012–16 all cancer almost caught up: 1-survival for cervical cancer was 88% compared to all cancer, 81–83%; 5- year survival 70% compared 64–67% for all cancer.

We calculated a difference between 1- and 5-year survival as an indicator of how well survival was maintained between years 1 and 5 after diagnosis. For cervical cancer improvement was observed from 6 to 4% units for countries other than FI where no improvement was observed. For vaginal and vulvar cancer, no essential improvement could be observed (but the periodic fluctuations were large). For all cancers the improvements were DK 2% units, FI 7% units, NO 5% units and SE 3% units. Thus for all cancers, some success was achieved even between years 1 and 5, in agreement with cervical cancer, but even for them the main driver in positive survival development has been the gains in 1-year survival. We have carried out this kind of analysis on some other cancers in FI and SE [46–48]. In hematological malignancies and rectal cancer respectable progress has been made in survival between years 1 and 5, whereas for colon and kidney cancer progress has been nil probably indicating that metastatic growth is difficult to contain [46–48].

The limitations of the study are that we have no clinical or risk factor data that would allow further stratification of the patients. However, over 80% of these cancers are squamous cell carcinomas, and in the age-incidence relationships stage does not differ between the main type and adenocarcinoma [25]. Stage data are lacking in NORDCAN which is a disadvantage for survival studies. As discussed above, screening has been most effective in detecting advanced stage cervical cancer. Historical data (90 years) from a large Stockholm hospital show that stage III and IV cervical cancers were close to their present level (< 20% of all cases) already in the 1960s, and that the main influence of screening was an increase in stage I (> 60%) and a decrease in stage II (< 20%) disease [18, 49]. Such long-term data are not available at the national level but the shift between stage I and II cancer is also documented in the nationwide Swedish cohort study [33]. This change is most likely contributing to the survival improvement [18]. The strengths are long-term high-quality data from four national cancer registries.

In conclusion, we observed country specific decline in the incidence of cervical cancer which was coincident with rolling out of screening activities. The attained plateau incidence was lowest at 4/100,000 in FI and highest at 10/100,000 in DK and NO. The incidence of vaginal and vulvar cancer remained relatively constant at about 2/100,000. Relative 1-year survival in cervical cancer improved identically for all countries from low 80%s to high 80%s in the 50-year period, and 5-year survival improved also but at 20% unit lower level. Survival in vaginal and vulvar cancer followed the same patterns but at a few % units lower level. Considering that limited improvement in survival was observed between years 1 and 5 and that no improvement in survival was evident among patients diagnosed past the screening age, the study fails to find evidence that treatment, other than local excision triggered by screening, would have contributed to the survival improvements over the 50-year period. As screening and largely also treatment appear to have reached their limits, the fight against cervical, vaginal and other HPV-related cancer is now relying on HPV vaccination which may have global preventive perspectives which screening was not able to fulfill [39–41, 50, 51]. With regard to treatment of metastatic cervical cancer, immunotherapy appears to provide a much-needed increase in efficacy [52, 53]. The presence of foreign viral proteins resulting from HPV infection facilitates use of immunotherapy for treatment of these tumor types. For vulvar cancer, HPV infections are diagnosed mostly in young patients and novel therapeutic approaches are being sought for all patients, including immunotherapies [20].

## Data Availability

Publicly available NORDCAN data can be accessed at (https://NORDCAN.iarc.fr/en/database#bloc2).

## Abbreviations

DK: Denmark
FI: Finland
HPV: Human papilloma virus
NO: Norway
SE: Sweden
